# Prophylactic mesh reinforcement after open aortic aneurysm repair: a prospective cohort study

**DOI:** 10.3389/fsurg.2025.1658180

**Published:** 2025-11-14

**Authors:** Raffaella Sguinzi, Melissa Lagger, Théo Chevalley, Benoît Gremaud, Markus Menth, Leo Buhler, Michel Adamina

**Affiliations:** 1 Department of Surgery, Fribourg Cantonal Hospital, Villars-sur-Glâne, Switzerland; 2 Department of Medical and Surgical Specialties, Faculty of Science and Medicine, University of Fribourg, Fribourg, Switzerland; 3 Department of Visceral Surgery and Medicine, Inselspital, Bern University Hospital, Bern, Switzerland

**Keywords:** abdominal aortic aneurysm (AAA), prophylactic mesh reinforcement, incisional hernia, retromuscular mesh, vascular surgery outcomes

## Abstract

**Objectives:**

Patients undergoing elective open abdominal aortic aneurysm (AAA) repair via midline laparotomy are at significantly increased risk—up to threefold—of developing incisional hernias (IHs) compared to those treated for aorto-iliac occlusive disease using the same approach. Recent vascular surgery guidelines recommend prophylactic mesh reinforcement (PMR) during abdominal wall closure to reduce IH incidence. This study aims at evaluating the effectiveness of retromuscular PMR in preventing IH after open AAA repair and to assess related postoperative complications.

**Methods:**

This was a prospective cohort study including patients who underwent open AAA repair with retromuscular PMR at our institution. Data collection included patient demographics, operative details, and postoperative complications. Clinical examination, abdominal ultrasound, and quality of life (QoL) were routinely assessed to evaluate the presence of IH and patient-reported outcomes. The primary endpoint was the incidence of IH; secondary outcomes included fascial dehiscence, seromas, surgical site infections (SSI), hematomas, chronic pain, and mesh displacement. Descriptive statistics were used to report outcomes, and findings were compared with existing literature.

**Results:**

A total of 21 patients were included between 2019 and 2024 with a median follow-up of 32 months. IH occurred in 4 (19%) patients: three developed hernias after a re-laparotomy performed postoperatively with mesh incision and re-closure, and one hernia was detected on ultrasound without clinical symptoms. No cases of fascial dehiscence, seroma, or surgical site infection were reported, and nor was chronic pain or mesh displacement. QoL was well-preserved, with minimal functional limitations and an average general health score of 80%.

**Conclusions:**

Retromuscular PMR may reduce the incidence of IH after open AAA repair. Re-laparotomy appears to be a risk factor for hernia development. Although these results support current guideline recommendations, further data with larger cohorts are needed to confirm these findings.

**Registration number:**

Observational study NCT06762561 (https://www.clinicaltrials.gov).

## Introduction

Patients undergoing open abdominal aortic aneurysm (AAA) repair via a midline laparotomy face a nearly threefold increased risk of developing an incisional hernia (IH) when compared to patients treated for aorto-iliac occlusive disease with the same incision. IH is associated with a marked reduction in quality of life (QoL), a high incidence of re-intervention, and substantial risk (1–5).

Regarding the general principles of the midline laparotomy closure technique, the European and the American Hernia Societies recommend the use of a prophylactic mesh in high-risk patients (including diabetes, smoking, chronic pulmonary obstructive disease [COPD], obesity, immunosuppression and surgical site infection (6–9)), without providing specific guidelines for emergency procedures (10). For vascular surgery, guidelines from the European Society of Vascular Surgery recommend prophylactic mesh reinforcement (PMR) for the abdominal closure (11). Indeed, multiple randomized controlled trials (12–18) and meta-analyses (19–21) have demonstrated a consistent effect of PMR in prevention of IH, particularly in high-risk patients undergoing AAA repair. Furthermore, mesh-related complications, such as infections or seromas, do not appear to be significantly increased in patients who received mesh augmentation for this indication (12–18).

This study aims to evaluate the implementation of current vascular surgery guidelines on prophylactic mesh use for abdominal wall closure and to assess the safety and clinical benefits of retromuscular mesh reinforcement in patients undergoing open AAA repair at our institution.

## Materials and methods

### Study design

This is a single-center, prospective cohort study conducted at the Fribourg Cantonal Hospital. In this institution, all vascular surgeries are entered in Swissvasc, the clinical registry of the Swiss Society of Vascular Surgery. Swissvasc was developed in accordance with the VASCUNET template with regular independent auditing and high data quality. Institutional volume, risk factors, quality of indication, and in-hospital outcomes at discharge are published yearly (22).

### Sample size

In our institution, we perform approximately 5–10 open elective AAA repairs per year but we do not perform emergency AAA repairs. Our sample includes all consecutive eligible patients over a 5-year period (2019–2024), with a total of 21. This approach ensured inclusion of a complete, unselected cohort, thereby enhancing the internal validity of our findings despite the limited statistical power.

### Origin and management of data

Clinical data from medical patient records, operative reports, and postoperative follow-up were routinely entered in the Swissvasc registry. A subset of data was extracted for the purpose of the present study, including patients' demographics and key risk factors such as body mass index (BMI), alcohol consumption, tobacco use, COPD, diabetes, immunosuppression, and history of previous laparotomy. The present study was reviewed and approved by the Cantonal Ethical Board (CER-VD 2025-00171).

### Inclusion criteria

This study included all adult patients who underwent elective open AAA repair with prophylactic mesh reinforcement at Fribourg Cantonal Hospital between 2019 and 2024. All participants consented to participate in the present study.

### Exclusion criteria

Patients who underwent emergency open AAA repair or endovascular AAA repair were excluded.

### Outcomes

The primary outcome of this study was the effectiveness of PMR in reducing the incidence of IH after elective AAA repair.

Secondary outcomes were the assessment of postoperative complications, such as surgical site infections, seroma formation, hematomas, fascial dehiscence, as well as operative time and QoL.

#### Patient timeline

Patients were followed postoperatively for at least 1 year after AAA repair. At the 1-year postoperative consultation, clinical examination by a vascular surgeon and abdominal ultrasound (US) by the same experimented consultant radiologist were performed, both specifically evaluating the abdominal wall. Whenever the US was inconclusive, a computed tomography (CT) scan was performed. When an incisional hernia was diagnosed, patients were counselled regarding potential surgical management options. Finally, patient-reported outcomes were assessed using the validated EQ-5D-5l, a standardized measure of health-related quality of life (23, 24) (Appendix 1).

#### Surgical technique

After completing the open AAA repair, the focus shifted to abdominal wall closure, following the technique outlined below. The rectus abdominis muscles were mobilized by carefully dissecting the muscle fibers and separating them from the posterior rectus sheath to create a retromuscular space. The posterior rectus sheath was then closed with a continuous suture of slow-absorbable PDS 2/0. A Medtronic™ Progrip™ Preventive Self-Gripping Polyester mesh or an Ultrapro mesh™ was placed in the retromuscular plane without tension, covering the retrorectus sheath for 4 cm on each side from the midline. Mesh selection was based on intraoperative assessment of abdominal wall dimensions and surgeon preference, within a standardized mesh placement protocol. The mesh was secured to the lower abdominal wall with four points of Prolene 2/0 to anchor the mesh at its corners to the lower abdominal wall. This technique was chosen to prevent mesh migration while minimizing foreign body load and operative time. The anterior rectus sheath was closed using a continuous PDS 2/0 suture with small bites. No drainage, antibiotics, or abdominal binders were used as part of the protocol.

#### Abdominal ultrasound

**Figure 1:**
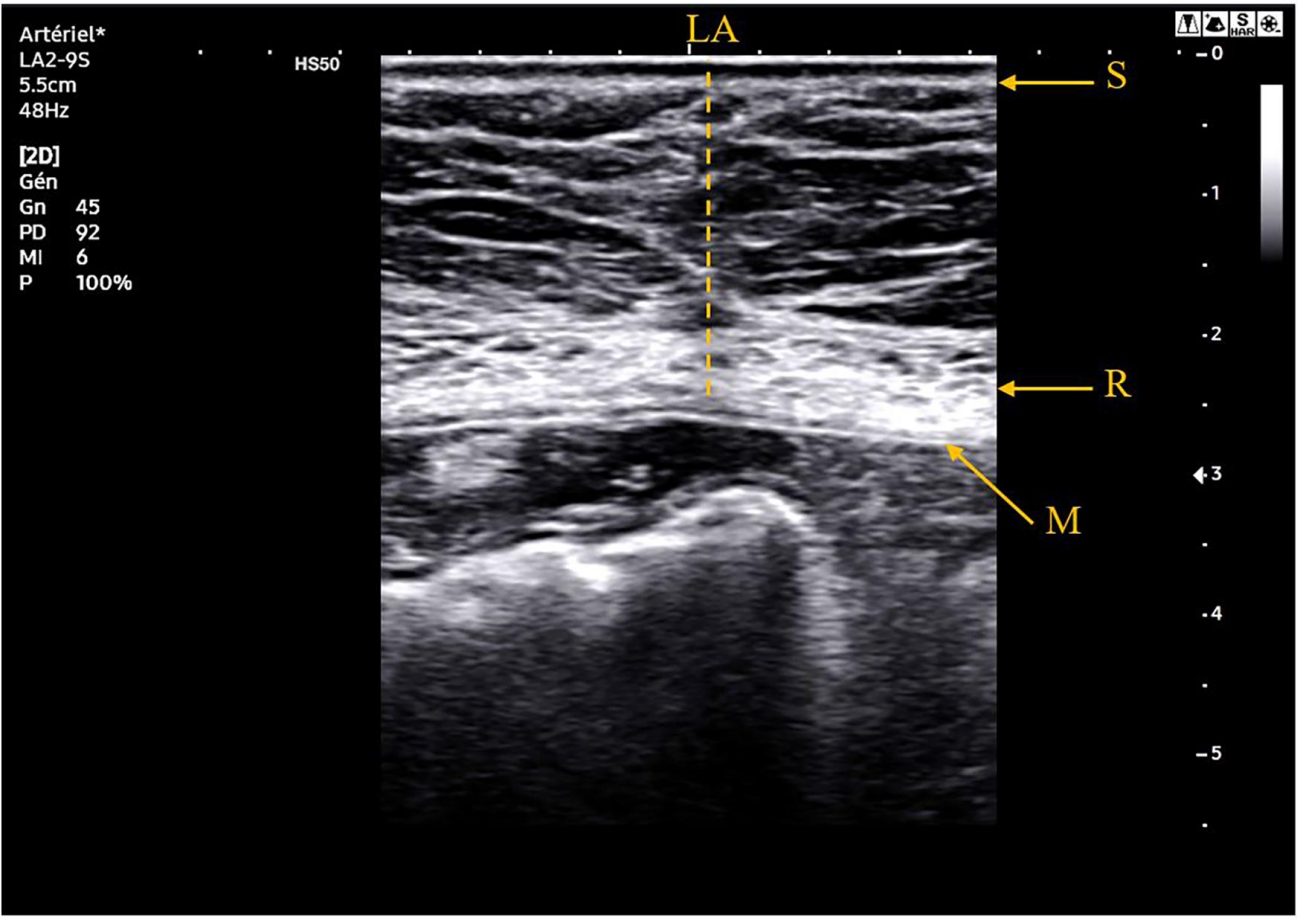
Ultrasound image at 78 months of follow-up of a 76-year-old patient who did not present any incisional hernia. S, skin; R, rectus abdomini muscle; LA, linea alba; M, retromuscular mesh.

The ultrasound was performed using a defined protocol by a Samsung Medison HS50® with a convex probe (CA1-7AD®) for studying the aorta and a linear probe (LA2-9S®) for examining the abdominal wall and visualizing the mesh. The mesh appears as a retromuscular hyperechoic image. A normal ultrasound examination is shown in Figure 1.

#### Statistical analysis

R Statistical Software has been used to ensure transparency and reproducibility. Descriptive statistics have been used to assess both the primary endpoint (incidence of incisional hernias) and secondary endpoints (postoperative complications). After assessing the normality of the data, we conducted a *t*-test for quantitative data and Fisher’s exact test for categorical data. The observed rate of incisional hernias has been reported as a percentage and compared to literature-reported rates for non-mesh cases. For secondary endpoints, descriptive statistics summarized complication rates and risk factors with median and standard deviations. Results will be compared with findings from the existing literature. A *p*-value <0.05 was considered significant.

## Results

**Figure 2:**
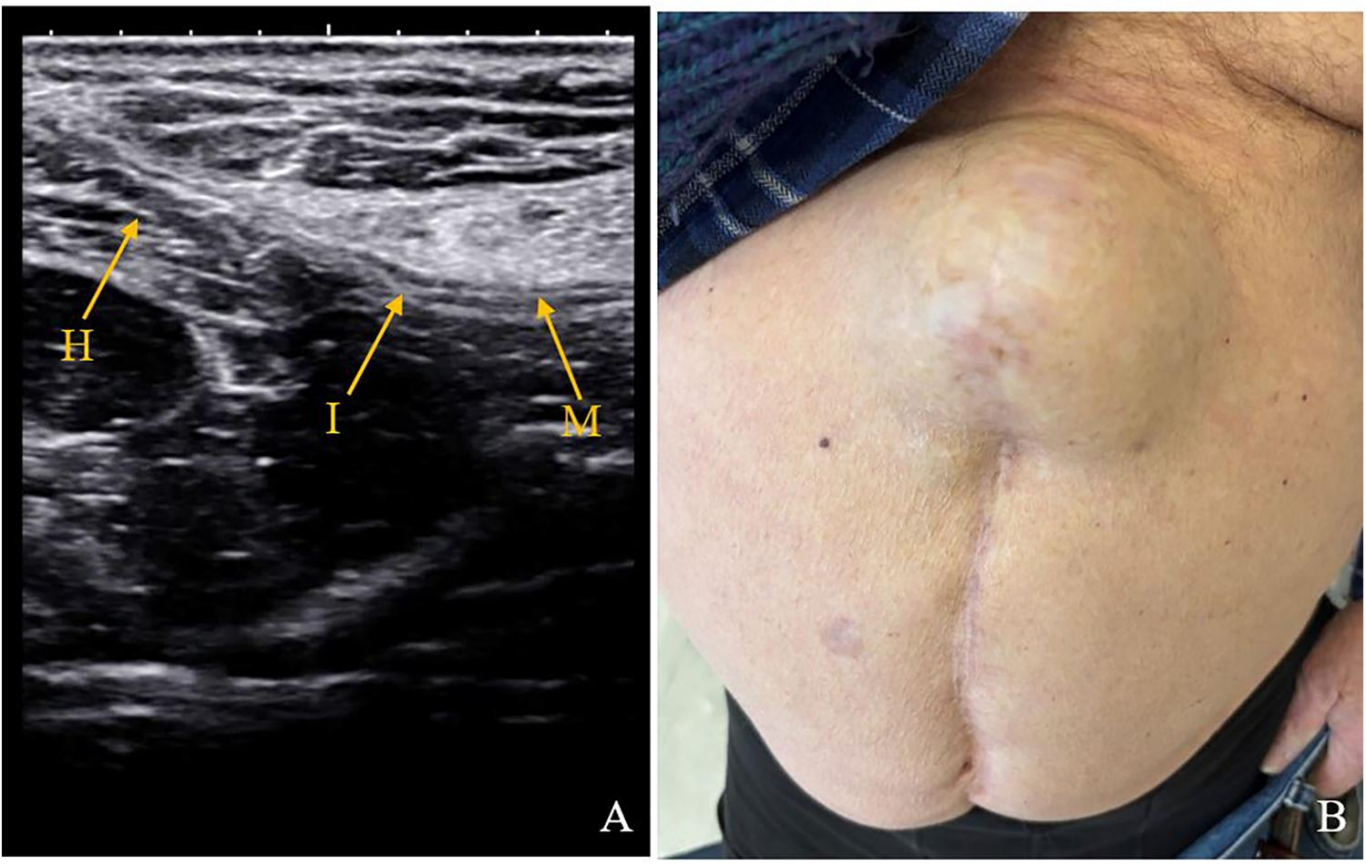
Ultrasound and clinical images of an 83-year-old patient who underwent re-laparotomy on day 13th postoperatively for hemo-peritoneum. **(A)** Ultrasound image at 23 months post operatively with incisional hernia. **(B)** Clinical image of a 8 cm incisional hernia. M, mesh; I, mesh interruption; H, hernia.

**Figure 3:**
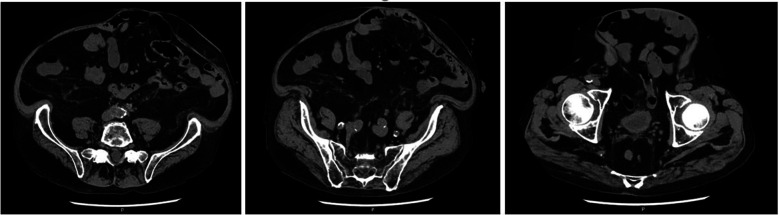
CT scan images at 4 years follow-up of a 71-year-old patient who underwent re-laparotomy 1 month after AAA repair for an ischemic sigmoid perforation and developed a loss of domain hernia due to a severe would infection.

**Figure 4:**
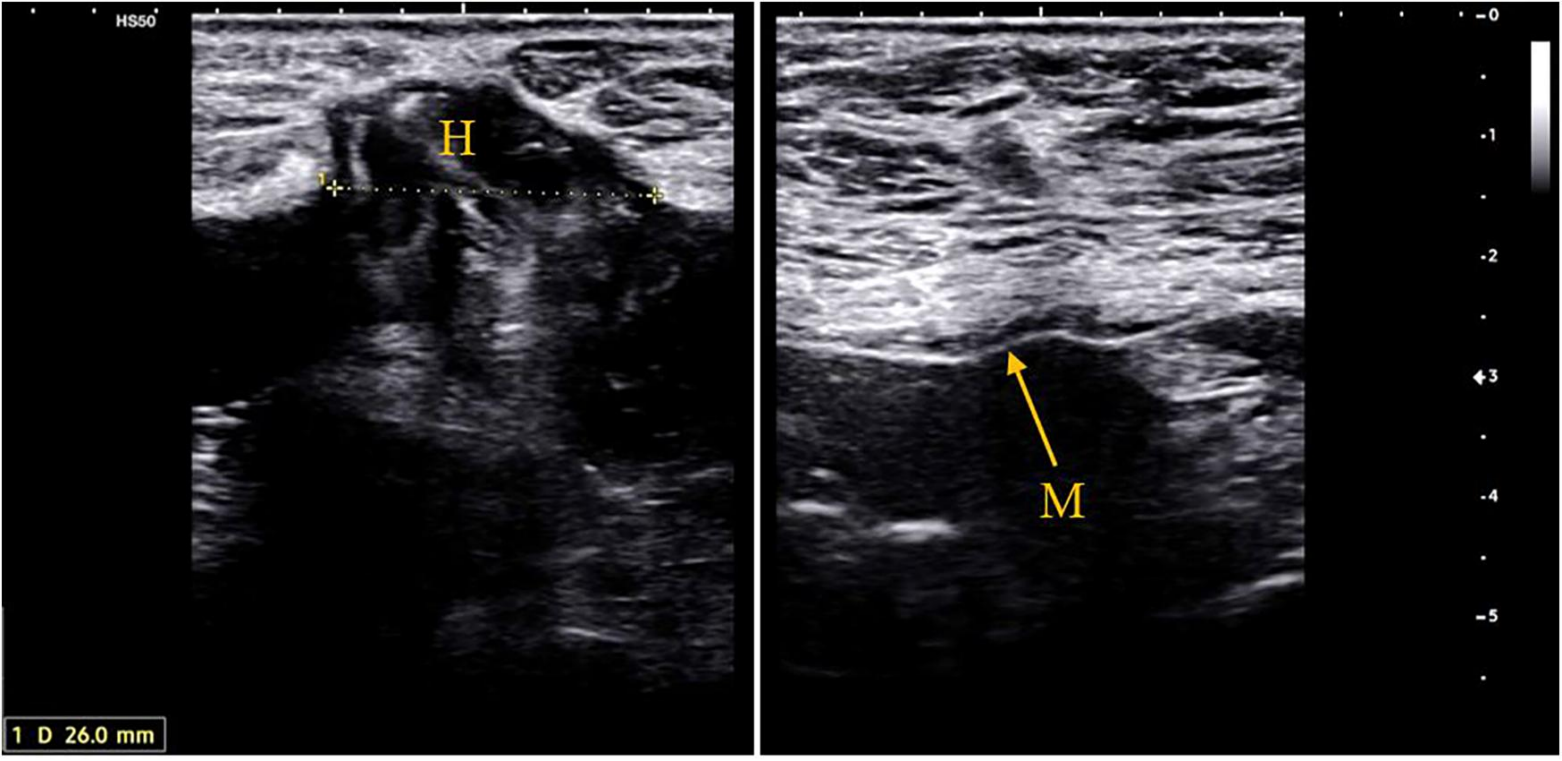
Ultrasound images at 43 months follow-up of a 70-year-old patient who underwent re-laparotomy 18 months after AAA repair for an adenocarcinoma of the gastroesophageal junction. **(A)** 26 mm. supra-umbilical hernia. **(B)** Ultrasound image showing the retro-muscular mesh in place M, mesh; H, hernia.

A total of 21 patients who underwent open aortic aneurysm repair with prophylactic mesh reinforcement were included in this prospective cohort study. The median follow-up duration was 32 months (interquartile range 19–48). Patients' demographical characteristics and risk factors are described in Table 1. The overall incidence of IH was 19% (4/21); no patients developed fascial dehiscence, seromas, or surgical site infections. Patients were followed for a median of 32 months, during which no mesh-related long-term adverse events, including chronic pain or mesh displacement, were clinically reported or detected on follow-up imaging. Moreover, QoL was specifically assessed using the EQ-5D-5l, which includes pain and discomfort dimensions, and showed preserved QoL in the cohort (Table 2). Patient-reported quality of life was well-preserved, with the majority experiencing minimal to no functional impairment; the average general health score was 80% (79% for men and 85% for women), reflecting favorable postoperative outcomes (Table 3). Notably, two IHs occurred in patients who had undergone subsequent re-laparotomy <6 months postoperatively, with incision and subsequent closure of the mesh. The first of those patients was an 83-year-old man who underwent re-laparotomy on postoperative day 13 for hemoperitoneum with no active bleeding. The mesh was incised during surgery and closed with Prolene 0. He developed an 8 cm sub-xiphoidal incisional hernia (Figure 2). The second patient was a 71-year-old man who presented an ischemic sigmoid perforation 1 month postoperatively. He underwent a sigmoidectomy and placement of an end colostomy (Hartmann's procedure). The grossly contaminated surgical field mandated partial removal of the mesh. A subsequent wound infection was treated with negative pressure dressing. The patient then developed a major IH with loss of domain (Figure 3). The third patient was a 70-year-old man who underwent re-laparotomy 18 months after AAA repair for an adenocarcinoma of the gastroesophageal junction. Again, the mesh was incised and subsequently closed with PDS 0 loop sutures. He developed a 2.6 cm supra-umbilical hernia that could be appreciated clinically and was confirmed by US (Figure 4). The fourth patient with IH was identified incidentally through US during an uneventful follow-up.

**Table 1:** Patients' demographical characteristics expressed as number (%) or mean ± SD.

Characteristic	Overall, *N* = 21
Males	17 (80)
Age	69.6 ± 9.6
BMI	26.3 ± 3.8
Tabaco	
Active	10 (47)
Ancient	9 (43)
Never	2 (10)
UPA median, mean	41.2 ± 24
Alcohol	
Occasionally	12 (57)
1 glass/day	5 (24)
>1 glass/day	4 (19)
BPCO	2 (10)
Diabetes	2 (10)
Immunosuppression	0 (0)
ASA score	
I	0 (0)
II	5 (24)
III	13 (62)
IV	3 (14)
Previous laparotomy	1 (5)

**Table 2:** Outcomes at median follow-up of 32 months expressed as number (%) or mean (+/−SD).

Outcomes	Overall (*n* = 21)	<6 months postoperative re-laparotomy (*n* = 2)
Incisional hernia	4 (19)	
Clinical and US	3 (14)	2 (100)
US only	1 (5)	2 (100)
Fascial dehiscence	0 (0)	0 (0)
Seroma	0 (0)	0 (0)
Surgical site infection	0 (0)	0 (0)
Hematoma	0 (0)	0 (0)
Other postoperative complications		
Postoperative ileus	5 (24)	2 (100)
Pulmonary embolism	2 (10)	1 (50)

**Table 3:** EQ-5D-5l questionnaire results expressed as number (%).

	Male 17 (80)	Female 4 (20)	Total
Mobility			
I have no problems in walking about	13 (76)	4 (100)	17 (80)
I have slight problems in walking about	3 (17)	0 (0)	3 (14)
I have moderate problems in walking about	1 (5)	0 (0)	1 (5)
I have severe problems in walking about	0 (0)	0 (0)	0 (0)
I am unable to walk about	0 (0)	0 (0)	0 (0)
Self care			
I have no problems washing or dressing myself	15 (88)	4 (100)	19 (90)
I have slight problems washing or dressing myself	2 (11)	0 (0)	2 (10)
I have moderate problems washing or dressing myself	0 (0)	0 (0)	0 (0)
I have severe problems washing or dressing myself	0 (0)	0 (0)	0 (0)
I am unable to wash or dress myself	0 (0)	0 (0)	0 (0)
Usual activities			
I have no problems doing my usual activities	13 (76)	4 (100)	17 (80)
I have slight problems doing my usual activities	3 (17)	0 (0)	3 (14)
I have moderate problems doing my usual activities	1 (5)	0 (0)	1 (5)
I have severe problems doing my usual activities	0 (0)	0 (0)	0 (0)
I am unable to do my usual activities	0 (0)	0 (0)	0 (0)
Pain/discomfort			
I have no pain or discomfort	15 (88)	4 (100)	19 (90)
I have slight pain or discomfort	2 (11)	0 (0)	2 (10)
I have moderate pain or discomfort	0 (0)	0 (0)	0 (0)
I have severe pain or discomfort	0 (0)	0 (0)	0 (0)
I have extreme pain or discomfort	0 (0)	0 (0)	0 (0)
Anxiety/depression			
I am not anxious or depressed	14 (82)	4 (100)	18 (85)
I am slightly anxious or depressed	2 (11)	0 (0)	2 (10)
I am moderately anxious or depressed	0 (0)	0 (0)	0 (0)
I am severely anxious or depressed	0 (0)	0 (0)	1 (5)
I am extremely anxious or depressed	0 (0)	0 (0)	0 (0)
Mean general health score on visual analogic scale (%)	79 ± 13	85 ± 7	80 ± 12

Patient-reported outcomes were measured and compared to an age- and gender-matched population of reference. As no Swiss population data are available and the vast majority of our study population spoke French, health-related quality-of-life norm values from the French population were used. Hence, a French reference value for the patient's self-rated health on a visual analog scale (VAS) was used, with 0 indicating the worst health and 100 indicating the best health an individual could imagine. The French population reported an age-matched VAS of 74 for men and of 71.9 for women (72.9 for men and women together), which was 10% lower than in our study population. Interestingly, the age- and gender-matched VAS of Americans was similar to the QoL value reported by our study population (25, 26).

## Discussion

Since 2019, our institution has routinely implemented PMR in patients undergoing open AAA repair. As part of our standardized follow-up protocol, all patients underwent a combined clinical and ultrasound examination at 1 year postoperatively to assess for incisional hernia formation. During the same follow-up visit, patients were also asked to complete the EQ-5D quality-of-life questionnaire, a validated instrument commonly used in health outcome research. This integrated follow-up approach allowed for a comprehensive evaluation of both clinical outcomes and patient-reported quality of life, supporting a broader assessment of the value of PMR in complex abdominal surgery.

A cost-utility analysis by Fischer et al. (27) demonstrated that prophylactic mesh reinforcement is not only more effective than primary suture closure in preventing IH, but also more cost-effective by offering better clinical outcomes at a lower total cost to the healthcare system compared to primary suture closure.

Although prophylactic mesh use offers benefits, its application in contaminated or infected surgical fields poses significant risks, including mesh infections, abscesses, and mesh rejection (28–30). Current guidelines recommend caution in patients with infection risks, as complications may outweigh the potential benefits of mesh reinforcement, thereby emphasizing the importance of PMR in laparotomy for elective AAA repair.

The choice of a Progrip™ Preventive Self-Gripping Polyester mesh was driven by the inherent quality of a polyester mesh for sublay placement and by its dimensions. Indeed, its width of 8 cm was ideal for retromuscular placement, while its length, in the range of 10–40 cm, made it very suitable for xipho-pubic laparotomies, such as in cases of aneurysms. In addition, it does not require many fixation sutures. The choice of a retromuscular location for the mesh minimized direct contact between the mesh and the peritoneal cavity, reducing the risk of complications, such as adhesions or infections. Indeed, two meta-analyses and a dedicated risk–benefit assessment (31, 32) found that a sublay placement provided superior results in terms of IH prophylaxis and mesh-related complications when compared to other mesh positions. A third analysis specifically compared onlay to sublay placement of mesh and came to the same conclusion (33).

With regard to risk factors, re-laparotomy appeared to be a relevant risk factor for hernia development, as reported in other studies. Indeed, three of the four IHs observed in our cohort occurred in patients who had undergone subsequent reoperations, during which the prophylactic mesh had to be incised and later re-sutured. This finding raises important concerns regarding the durability of mesh protection after re-intervention. Evidence from the PRIMA trial's long-term follow-up (34) suggested that, although PMR generally led to lower rates of mesh-related complications, reoperations through a mesh-reinforced abdominal wall may necessitate partial mesh explantation or re-fixation, potentially compromising its protective function. Moreover, a large retrospective cohort study by Rios-Diaz et al. (35) reported that reoperation through a previously prosthetic-reinforced abdominal wall was associated with increased rates of surgical complications and healthcare utilization, suggesting that the presence of mesh may increase the complexity of subsequent surgical interventions. These observations underline the need for careful surgical planning during reoperations in patients with prophylactic mesh, and they suggest that disruption of the mesh integrity may reduce its efficacy in hernia prevention.

An important finding was that patients who were subjected to open AAA repair had a good quality of life at 1 year postoperatively, compared to the French general population and similar to the American population. When comparing QoL across populations, it is important to consider that a study sample is, by definition, different from the general population. Here, our study population was made of patients who were assessed 1 year after elective open AAA repair.

The present study has some limitations. The study's modest sample size limits the ability to draw causal conclusions, and the exclusion of emergency open AAA repairs leaves unaddressed a patient group potentially at highest risk for incisional hernia. However, the inclusion of consecutive patients from a dedicated, independently audited clinical registry ensures the reliability of real-world data and outcomes. Designed as a hypothesis-generating observational study rather than a confirmatory trial, no formal *a priori* power calculation was conducted due to its exploratory purpose. Our primary goal was to describe clinical outcomes using standardized follow-up and quality-of-life assessments in a patient population for whom current evidence supports PMR. We analyzed the observed hernia rate, complication profile, and patient-reported outcomes in the context of existing literature, taking care to avoid overgeneralizing our findings. The study specifically focused on elective AAA repairs, which offer a more standardized surgical and postoperative environment. Emergency AAA repairs, often complicated by hemodynamic instability, infection, or bowel ischemia, involve substantially different conditions that may necessitate altered surgical strategies, such as rapid closure or the inability to safely place a mesh, and are associated with a higher risk of postoperative complications, including incisional hernias. As such, our results cannot be extrapolated to emergency AAA repairs, highlighting the need for future research targeting this high-risk subgroup where evidence remains scarce.

The predominance of male patients (80%) in our cohort reflects the known epidemiology of AAA, which is significantly more common in men. However, this gender imbalance limits the generalizability of our findings to female patients. Due to the small number of women included in the study, meaningful subgroup analysis by sex was not feasible. Further studies with larger, more gender-balanced populations are necessary to explore potential sex-specific differences in outcomes and quality of life after PMR.

Our relatively small sample size also constrained the ability to perform stratified analyses based on specific reoperation causes (e.g., bleeding, infection) or to evaluate the influence of different surgical techniques on incisional hernia development. In addition, the limited number of incisional hernia events led us to adopt a descriptive approach when examining risk factors such as BMI and smoking, as conducting multivariate regression analyses could have resulted in overfitting and insufficient statistical power.

The absence of a control group in our study means that observed outcomes, particularly the incidence of incisional hernia and complication rates, were compared against published data from open AAA repairs without PMR. Variability in patient characteristics, risk profiles, surgical methods, and follow-up durations across these studies may limit direct comparability.

Our study also does not permit direct comparison between different mesh types or fixation techniques, which could represent a confounding factor.

The use of French EQ-5D-5l normative values for quality-of-life comparisons was selected based on linguistic and regional proximity, though cross-national comparisons may carry inherent limitations. Consequently, these results should be interpreted cautiously. Further research incorporating country-specific normative data or matched control groups would allow more precise contextualization of quality-of-life outcomes.

In conclusion, larger multicenter prospective studies are needed to facilitate robust subgroup analyses and comparative evaluations, thereby enhancing the strength and generalizability of the findings.

## Data Availability

The raw data supporting the conclusions of this article will be made available by the authors, without undue reservation.
